# HLA-A2 and B35 Restricted Hantaan Virus Nucleoprotein CD8^+^ T-Cell Epitope-Specific Immune Response Correlates with Milder Disease in Hemorrhagic Fever with Renal Syndrome

**DOI:** 10.1371/journal.pntd.0002076

**Published:** 2013-02-28

**Authors:** Ying Ma, Jiuping Wang, Bin Yuan, Meiliang Wang, Yun Zhang, Zhuwei Xu, Chunmei Zhang, Yusi Zhang, Bei Liu, Jing Yi, Kun Yang, Angang Yang, Ran Zhuang, Boquan Jin

**Affiliations:** 1 Department of Immunology, the Fourth Military Medical University, Xi'an, China; 2 Department of Infectious Diseases of Tangdu Hospital, the Fourth Military Medical University, Xi'an, China; University of Rhode Island, United States of America

## Abstract

**Background:**

Hantaan virus (HTNV) infection in humans is a serious public health concern in Asia. A potent T cell activation peptide vaccine from HTNV structure protein represents a promising immunotherapy for disease control. However, the T cell epitopes of the HTNV restricted by the HLA alleles and the role of epitope-specific T cell response after HTNV infection remain largely unexplored.

**Methodology/Principal Findings:**

Five well-conserved novel CD8^+^ T-cell epitopes of the HTNV nucleoprotein restricted by the most popular HLA alleles in Chinese Han population were defined with interferon-γ enzyme-linked immunospot assay in 37 patients infected with HTNV during hospitalization. Two epitopes aa129–aa137 and aa131–aa139 restricted by HLA-A2 and B35, respectively, were selected to evaluate the epitope-specific CD8^+^ T-cell response. HLA-peptide pentamer complex staining showed that the frequency of single epitope-specific CD8^+^ T cell could be detected in patients (95% confidence interval for aa129–aa137: 0.080%–0.208%; for aa131–aa139: 0.030%–0.094%). The frequency of epitope-specific pentamer^+^ CD8^+^ T-cell response was much higher in mild/moderate patients than in severe/critical ones at the acute stage of the disease. Moreover, the frequency of epitope-specific CD8^+^ T cells at acute stage was inversely associated with the peak level of serum creatinine and was positively associated with the nadir platelet counts during the hospitalization. The intracellular cytokine staining and the proliferation assay showed that the effective epitope-specific CD8^+^ T cells were characterized with the production of interferon-γ, expression of CD69 and the strong capacity of proliferation.

**Conclusion/Significance:**

The novel HLA class I restricted HTNV nucleoprotein epitopes-specific CD8^+^ T-cell responses would be closely related with the progression and the severity of the disease, which could provide the first step toward effective peptide vaccine development against HTNV infection in humans.

## Author Summary

Hantaan virus (HTNV), the prototype of the Hantavirus genus, is a rodent-borne pathogen that causes human hemorrhagic fever with renal syndrome (HFRS) with a mortality rate of approximately 15% in Asia. Since effective prevention is not available currently and the non-specific symptoms at the early stage of the disease always lead to the delay of visiting to hospital or misdiagnosis, alternative vaccinations against HTNV are of priority to overcome the problem. We defined five novel HTNV nucleoprotein CD8^+^ T-cell epitopes restricted by the most popular HLA alleles in Chinese Han population. For the first time, we quantitated the HTNV epitope-specific CD8^+^ T-cell frequency during HTNV infection and evaluated the correlations between the CD8^+^ T-cell response and the different outcomes of the HFRS severity. We also found that effective HTNV nucleoprotein epitope-specific CD8^+^ T-cell responses were characterized by the interferon-γ secretion with a strong capacity of activation and proliferation. Our results add weight to understanding the important role of epitope-specific CD8^+^ T-cell responses in the disease control after acute zoonotic HTNV infections in humans and provide a rationale foundation to speed up the process of peptide vaccine development.

## Introduction

Hantaan virus (HTNV), the prototype member of the genus Hantavirus of the family *Bunyaviridae*, was first isolated in Korea in 1978 and can cause a severe disease of hemorrhagic fever with renal syndrome (HFRS) in humans [1]. The genus Hantavirus is naturally maintained in persistently infected rodents and can be transmitted to humans via the inhalation of aerosols [2], [3]. Recently, pathogenic Hantaviruses have emerged as an increasing threat to human health, as not only are they the causative agents of HFRS in Asia and Europe that are associated with HTNV, Seoul virus, Dobrava virus, and Puumala virus (PUUV) infections [4]–[6], but also they can induce the severe hantavirus pulmonary syndrome (HPS) in North and South America, which is caused by Sin Nombre virus (SNV) and Andes virus serotypes [7], [8]. Specifically, the outbreak news recently reported 6 cases of HPS among visitors to Yosemite National Park in California and two of the 6 cases were fatal. The HFRS is a fulminant infectious disease which is characterized by fever, hemorrhage, renal impairment, and thrombocytopenia [9]–[11]. More than 100,000 cases of HFRS are reported annually, with a case fatality rate of approximately 15%. China is a severe epidemic area, where the HFRS human cases account for 90% of the total global cases [12].

Several studies have suggested that the Hantavirus infection could induce a vigorous cellular immune response in humans [13], [14], including an expansion of the number of activated circulating CD8^+^ T cells [15]–[17], an increase in the CD8^+^/CD4^+^ T-cells ratio [16], the infiltration of CD8^+^ T lymphocytes in the kidney biopsies of PUUV-infected patients [18], and the high frequencies of virus-specific memory CD8^+^ T lymphocytes that exist for a long time after the HTNV or PUUV infection [19]–[21]. It was reported that the T cell responses targeting the epitopes on viruses restricted by particular HLA alleles could contribute to the different outcome of the clinical course in many virus infectious diseases [22]–[24]. Our previous research found that the general T cell responses specific for HTNV nucleoprotein (NP), which is highly immunogenic and conservative, might help reduce the risk of progression to acute renal failure [25]. However, whether the HTNV epitopes specific T-cell responses restricted by different HLA alleles would correlate with the clinical severity in HFRS is still unclear.

Three cytotoxic T cell (CTL) epitopes of the HTNV (76–118 strain)-NP, aa421–aa429, aa334–aa342 and aa12–aa20 restricted by HLA-A1, A2.1, and B51, respectively were identified a decade ago [19], [26]. Recently, we have reported three novel CTL epitopes on HTNV-NP, including aa197–aa205, aa245–aa253, and aa258–aa266, which were restricted by various HLA alleles, including A11, A24, and B7 [27]. In addition, HTNV-NP C-terminal polypeptides aa301–aa315, aa355–aa369, and aa415–aa429 could induce strong CD8^+^ T-cell responses [28]. Based on these, the overall aim of our experiments is to identify the novel HTNV epitopes restricted by the major HLA alleles in Chinese Han population and to investigate the function of HTNV epitope-specific T-cell responses, which will be helpful to establish a better program for the diagnosis and treatment of HFRS, and to develop an effective peptide vaccine against the HTNV.

## Methods

### Ethics statement

Written informed consent was obtained from each HFRS patient or their guardians under a protocol approved by the Institutional Review Board of the Tangdu Hospital and the Fourth Military Medical University. The research involving human materials was also approved by the Ethical Review Board of the University, and the related information was used anonymously.

### Participants

A total of 37 adults presenting to the doctors with symptoms of fever, hemorrhage, effusion, and renal abnormalities and who were prospectively identified as HFRS were enrolled in this study from the Departments of Infectious Diseases at Tangdu Hospital of the Fourth Military Medical University (Xi'an, China). The clinical diagnosis of HFRS was confirmed by the detection of specific immunoglobulin M (IgM) or IgG antibodies to HTNV in the patients' serum specimens. The severity degree of the HFRS disease could be classified as previously described [25]: (1) Mild patients were identified with mild renal failure without an obvious oliguric stage; (2) moderate for those with obvious symptoms of uremia, effusion (bulbar conjunctiva), hemorrhage (skin and mucous membrane), and renal failure with a typical oliguric stage; (3) severe patients with severe uremia, effusion (bulbar conjunctiva and either peritoneum or pleura), hemorrhage (skin and mucous membrane), and renal failure with oliguria (urine output, 50–500 ml/day) for ≤5 days or anuria (urine output, <50 ml/day) for ≤2 days; and (4) critical ones with ≥1 of the following symptoms during severe disease: refractory shock, visceral hemorrhage, heart failure, pulmonary edema, brain edema, severe secondary infection, and severe renal failure with either oliguria (urine output, 50–500 ml/day) for >5 days, anuria (urine output, <50 ml/day) for >2 days, or a blood urea nitrogen level of >42.84 mmol/L. The patients who had other kidney disease, diabetes, cardiovascular disease, hematological disease, autoimmune disease, viral hepatitis, and other liver diseases were excluded in this study. The number of patients with severity degree of mild, moderate, severe, and critical was 5, 12, 9, and 11, respectively. According to the clinical observations, the illness could be divided into five sequential stages: febrile, hypotensive, oliguric, diuretic, and convalescent. The phase within 8 days from the fever onset to early oliguric stage was usually defined as acute or early stage of the disease. The detailed characteristics of the patients enrolled in the present study were summarized in Table S1. Four healthy adult volunteers, two women and two men, were selected as normal control donors for the present study.

### Procedures and investigations undertaken (see Text S1)

#### Statistical analysis

Statistical analyses were performed using SPSS 11.5 (SPSS Inc., USA). The frequency of the CD8^+^ T cells was presented as a 95% confidence interval (CI_95%_). The comparison of frequencies between the two groups was made using nonparametric Mann–Whitney *U* test. Associations between epitope-specific CD8^+^ T-cell frequency and clinical parameters were based on Spearman correlation test. A two-tailed *P* value below 0.05 (*P*≤0.05) was considered statistically significant.

## Results

### Identification of CD8^+^ T-cell epitopes on HTNV-NP

**Figure 1:**
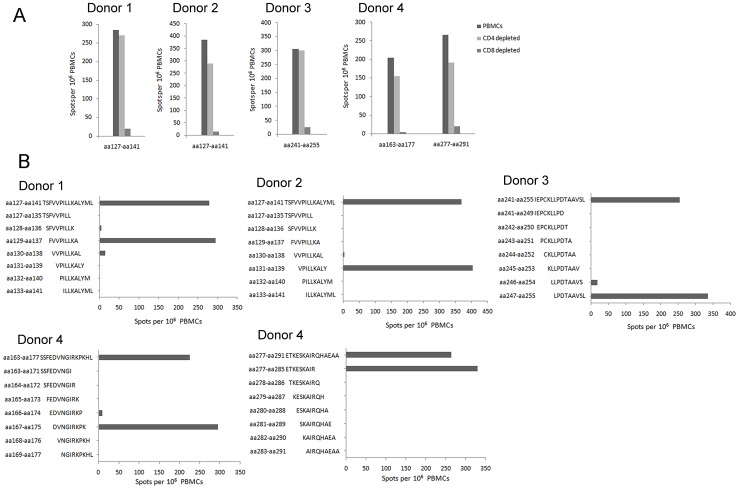
The identification of HTNV nucleoprotein CD8^+^ T-cell epitopes from HFRS patients. (A) The determination of effective epitope-specific T-cell responses. PBMCs from the four donors were depleted of CD4^+^ or CD8^+^ T cells, and stimulated with the indicated four 15-mer peptides, respectively. (B) Five novel nonamer peptides recognized by CD8^+^ T cells in the four donors were defined as HTNV nucleoprotein epitopes within the sequences of the 15-mer peptides nested inside. The *ex vivo* IFN-γ ELISPOT assay was performed. PBMC, peripheral blood mononuclear cell.

We identified five novel CD8^+^ T-cell epitopes on HTNV-NP from four HFRS patients through a similar investigation protocol as before [27]. The single-positive 15-mer peptides which could primarily stimulate the CD8^+^ T-cell response were screened out in Figure 1A, indicating that these peptides contained the CTL epitopes. The definition of the epitopes recognized by the CD8^+^ T cells showed that the aa129–aa137 (FVVPILLKA), aa131–aa139 (VPILLKALY), aa247–aa255 (LPDTAAVSL), aa167–aa175 (DVNGIRKPK), and aa277–aa285 (ETKESKAIR) were the HTNV-NP CTL epitopes that can simulate strong IFN-γ responses (Figure 1B).

### Analysis of the HLA restriction and the conservation of the CD8^+^ T-cell epitopes on the HTNV-NP

**Figure 2:**
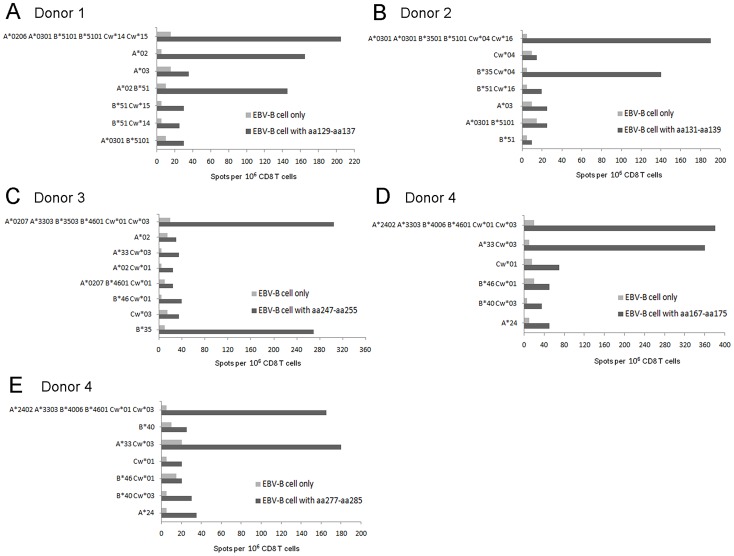
The analysis of HLA molecule restriction on CD8^+^ T-cell epitopes. The CD8^+^ T cell from donor 1 pre-sensitized with nonamer aa129–aa137 (A), donor 2 pre-sensitized with aa131–aa139 (B), donor 3 pre-sensitized with aa247–aa255 (C), and donor 4 pre-sensitized with aa167–aa175 (D) or aa277–aa285 (E) were tested against partially histocompatible EBV-B cells alone or pulsed with the particular peptide using *in vitro* IFN-γ ELISPOT assay. The HLA class I alleles shared between effectors and targets are indicated in ordinate. EBV-B, Epstein-barr virus-transformed B lymphoblast.

The *in vitro* peptide-specific pre-sensitized CD8^+^ T cells were successfully generated from all four donors, as defined by a flow cytometry analysis (data not show). The HLA-matched and mismatched EBV-B cells used to confirm HLA restrictions and the HLA class I molecules of the patients were shown in Figure 2. The nonamers recognized by these CD8^+^ T cells in the four donors were restricted by three different HLA class I alleles (Table 1). These novel nonamer epitopes were further supported by the binding motifs, mainly anchor residues at position 2 or position 9. The epitope aa129–aa137 fits the HLA-A2 binding motif at the anchor residues position 2 (leucine, valine or glutarnine). Epitopes aa131–aa139 and aa247–aa255 fit the HLA-B35 binding motif, including anchor residues at position 2 (proline, alanine, or valine) and the C terminus (leucine, tyrosine, or methionine). Epitopes aa167–aa175 and aa277–aa285 fit the HLA-A33 binding motif, including anchor residues at position 2 (alanine, isoleucine or valine) and the C terminus (arginine) [29]. When comparing HTNV to other Hantaviruses, the sequences of the epitopes aa129–aa137, aa131–aa139, and aa167–aa175 were conserved well with 67%–100% concordance among Hantaviruses, whereas the epitopes aa247–aa255 and aa277–aa285 were less well conserved (Table 2).

**Table 1:** The nonamer CD8^+^ T cell epitopes on the nucleoprotein of Hantaan virus and their HLA molecule restrictions.

Patients	Severity	15-mer peptide	Nonamer	Nonamer sequence	HLA Restriction	Frequency of pre-sensitized CD8^+^ T cells (SFC/10^6^ cells)
Donor1	severe	aa127–aa141	aa129–aa137	FVVPILLKA	HLA-A2	145–205
Donor2	critical	aa127–aa141	aa131–aa139	VPILLKALY	HLA-B35	140–190
Donor3	critical	aa241–aa255	aa247–aa255	LPDTAAVSL	HLA-B35	270–305
Donor4	mild	aa163–aa177	aa167–aa175	DVNGIRKPK	HLA-A33	360–380
		aa277–aa291	aa277–aa285	ETKESKAIR	HLA-A33	165–180

HLA, human leukocyte antigen; SFC, spot forming cell.

**Table 2:** The sequence conservation of Hantaan virus nucleoprotein CD8^+^ T-cell epitopes among Hantaviruses.

Virus	Strain	Epitope aa129–aa137	Epitope aa131–aa139	Epitope aa167–aa175	Epitope aa247–aa255	Epitope aa277–aa285	Genebank Accession No.
Old world							
Hantaan	76–118	FVVPILLKA	VPILLKALY	DVNGIRKPK	LPDTAAVSL	ETKESKAIR	M14626
Hantaan	Chen4	---------	---------	---------	---------	---------	AB027101
Hantaan	84FLi	---------	---------	---------	---------	---------	AF366568
Hantaan	A16	---------	---------	---------	---------	---------	AF288646
Dobrava	Dobrava	---------	---------	---------	-SEPSPT--	----AQ-V-	L41916
Seoul	SR-11	-----I---	---I-----	---------	MAESLIAGS	-P--FQ-L-	M34881
Seoul	R22	-----I---	---I-----	---------	MAESPIAGS	-P--FQ-L-	AF488707
Puumala	Sotkamo	-TL--I---	L--I-----	-I----R--	IKPEVKPGT	LD-NHV-DI	X61035
New world							
Sin Nombre	NM H10	-AL--I---	L--I-----	E-------R	--EQKDPRD	VSDIEDL-A	L25784
New York	RI-1	-AI--I---	I--I-----	E-------R	--EPRDPKD	VVDIEEL-V	U09488
Andes	Chile-9717869	-AI--I---	I--I-----	E--------	--KPKVA-E	VQDIIDL-D	NC_003466
Prospect Hill		-AI--I---	L--I----H	------R--	-KAEPRPGQ	LDETHLPDI	M34011

### Quantitation of HTNV-NP epitope-specific CD8^+^ T cells in peripheral blood mononuclear cell (PBMC) samples

**Figure 3:**
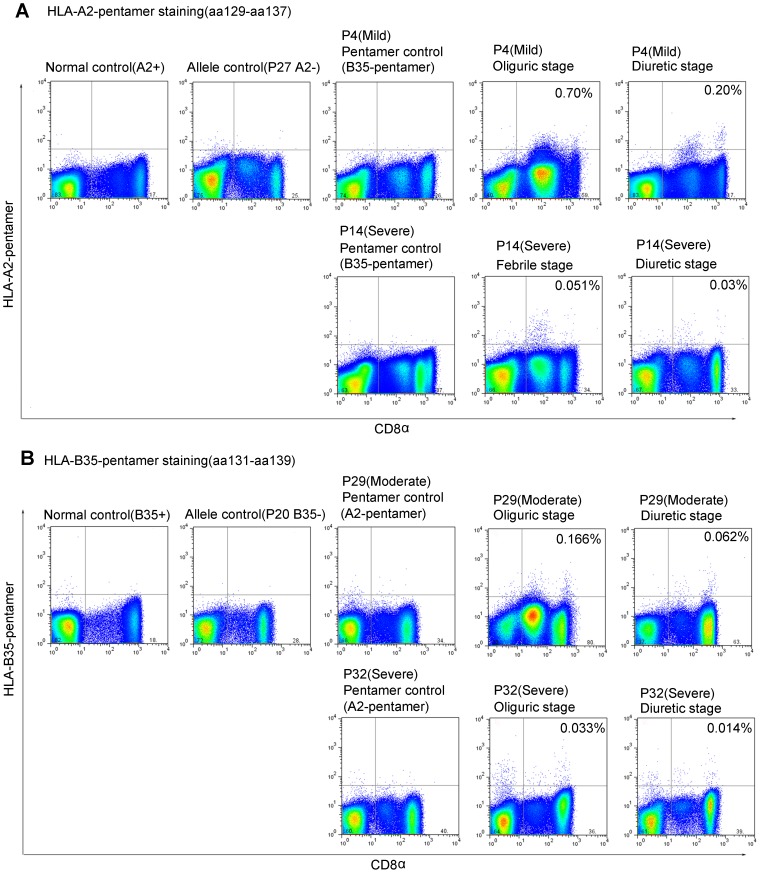
The accurate detection of HTNV nucleoprotein epitope-specific CD8^+^ T-cell response with HLA-peptide pentamer staining. (A) The PBMCs from HLA-A2^+^ donors P4 (mild) or P14 (severe) were stained with A2-pentamer preloaded of peptide aa129–aa137. (B) The PBMCs from HLA-B35^+^ donors P29 (moderate) or P32 (severe) were stained with B35-pentamer preloaded of aa131–aa139. Each of the donors has two time points, including febrile/oliguric and diuretic stages. A healthy person with HLA-A2 or B35 was stained as a normal control. The patient without HLA-A2 or B35 was used as an HLA allele control. The HLA-A2^+^ or B35^+^ patient stained with HLA-B35 or A2 pentamer, respectively, was used as a peptide control. HLA, human leukocyte antigen.

Since HLA-A2 is the most frequent allele (29.7%) of HLA-A loci in the Chinese Han population and HLA-B35 is a major allele in HLA-B loci [30], we focused the epitopes aa129–aa137 and aa131–aa139 restricted by HLA-A2 and HLA-B35, respectively, and generated the HLA class I peptide pentameric complex for these two HTNV-NP epitopes. Twenty-five HLA-A2^+^ patients and eight HLA-B35^+^ patients with a different severity of HFRS were tested at early and late time points during hospitalization. Epitope aa129–aa137-specific pentamer^+^ CD8^+^ T cells in PBMCs could be detected in 11 of the 12 (91.7%) patients with mild/moderate severity as compared with 7 of the 13 (53.8%) performed on severe/critical patients (Table S1). (Fisher's exact chi-square test, *P* = 0.073). The frequency of CD8^+^ T cells that were specific for the HLA-A2-restricted epitope aa129–aa137 ranged from 0.010 to 0.700% (CI_95%_: 0.080%–0.208%). For the HLA-B35^+^ patients, the frequency of the circulating CD8^+^ T cells that were specific for the epitope aa131–aa139 ranged from 0.010 to 0.185% (CI_95%_: 0.030%–0.094%) (Figure 3).

### HTNV-NP epitope-specific CD8^+^ T-cell responses associated with the outcome of the disease in HFRS patients

**Figure 4:**
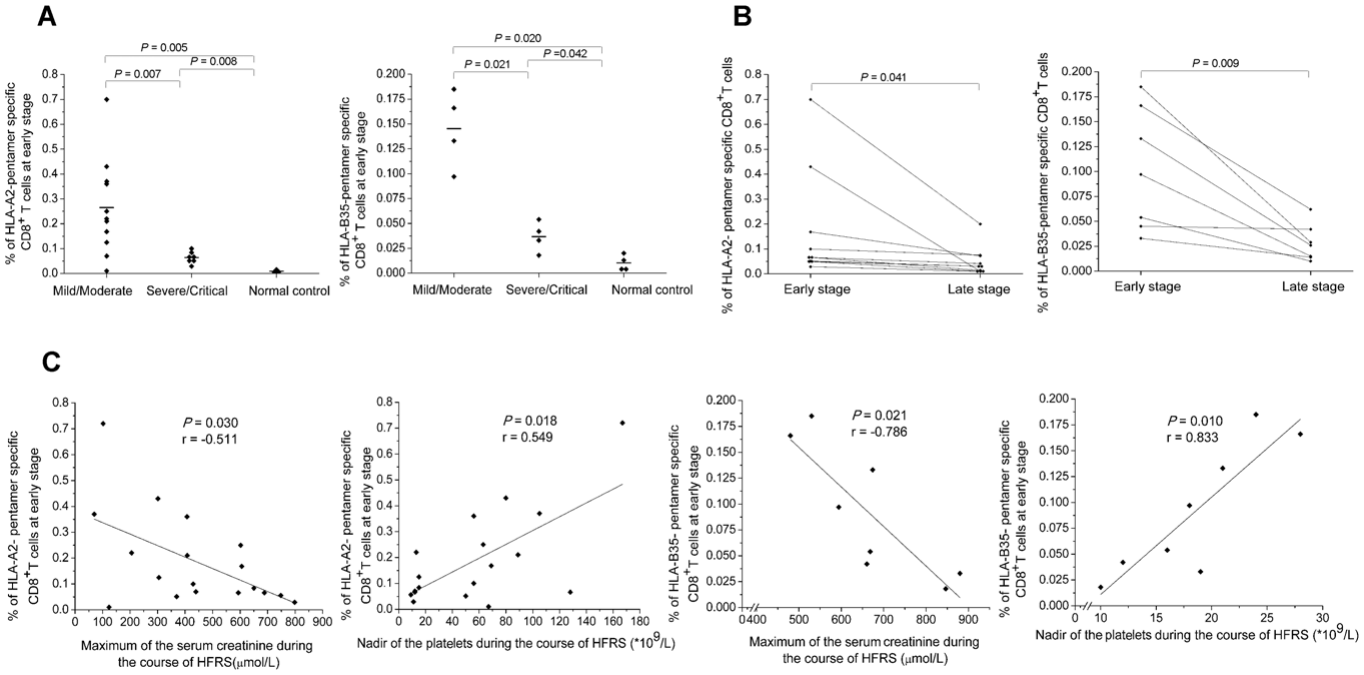
The analysis of HTNV nucleoprotein epitope-specific CD8^+^ T-cell responses with the disease severity. The comparison of the epitope-specific CD8^+^ T-cell frequencies (A) between patients in mild/moderate group and patients in severe/critical group at the acute stage in the 18 donors with HLA-A2 or in the 8 donors with HLA-B35, (B) between the acute stage and the late stage in ten HLA-A2^+^ patients and in seven HLA-B35^+^ patients (Mann-Whitney *U* test). (C) The associations between the frequencies of the epitope-specific CD8^+^ T cells at the acute stage and the peak level of serum creatinine or nadir platelet counts during the hospitalization in 18 donors with HLA-A2 and in 8 donors with HLA-B35 (Spearman correlation test). *P* values of ≤0.05 were considered statistically significant. HLA, human leukocyte antigen; HFRS, hemorrhagic fever with renal syndrome.

We compared the epitope-specific CD8^+^ T-cell frequencies between patients with mild/moderate HFRS and those with severe/critical HFRS at the earliest available acute stage time point in the 18 patients with HLA-A2 and in the 8 patients with HLA-B35 from whom PBMCs had been collected. The statistical analyses showed that the frequencies of the epitope-specific CD8^+^ T cells were much higher in the mild/moderate HFRS patients than that in the severe/critical patients (Mann–Whitney *U* test, for HLA-A2 restricted epitope: *P* = 0.007, for HLA-B35 restricted epitope: *P* = 0.021) (Figure 4A). There was no difference in the frequency between the two groups at the late stage of the disease (data not shown). Then we compared the frequencies of the epitope-specific CD8^+^ T-cell response between the acute stage and the late stage (diuretic and convalescence stage) during the illness in ten HLA-A2^+^ patients and in seven HLA-B35^+^ patients, and the results showed that the frequency of the epitope-specific CD8^+^ T cells in the acute stage was higher than that in the late stage in patients (Mann–Whitney *U* test, for HLA-A2 restricted epitope: *P* = 0.041, for HLA-B35 restricted epitope: *P* = 0.009) (Figure 4B). The analysis of the associations between the HTNV-NP epitope-specific CD8^+^ T-cell responses and the clinical parameters showed that the frequency of epitope-specific CD8^+^ T cells at the acute stage was inversely associated with the peak level of serum creatinine (Spearman correlation test, for HLA-A2 restricted epitope: *P* = 0.030, r = −0.511; for HLA-B35 restricted epitope: *P* = 0.021, r = −0.786) and positively associated with the nadir of platelet counts (for HLA-A2 restricted epitope: *P* = 0.018, r = 0.549; for HLA-B35 restricted epitope: *P* = 0.010, r = 0.833) during the clinical course of the HFRS (Figure 4C).

### Effector functions of HTNV-NP epitope-specific CD8^+^ T cells

**Figure 5:**
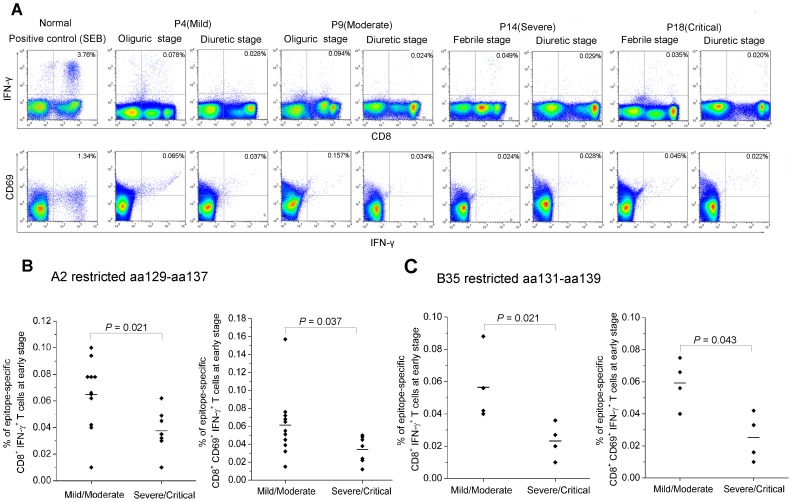
The frequency of IFN-γ produced by activated HTNV nucleoprotein epitope-specific CD8^+^ T cells in patients. (A) The intracellular cytokine staining assay was applied to detect the frequencies of epitope-specific IFN-γ production CD8^+^ T cells expressing the active marker CD69 in PBMCs from mild patient P4, moderate patient P9, severe patient P14, and critical patient P18. Each of the patients has two time points, including febrile/oliguric and diuretic stages. All the four patients are HLA-A2^+^ and stimulated with peptide aa129–aa137. FACS contour plots were gated on CD3^+^ CD8^+^ cells. Percentages of double-positive cells are shown. The healthy PBMCs stimulated with SEB (200 ng/ml) was used as a positive control. (B–C) The comparison of the frequency of epitope aa129–aa137 or aa131–aa139-specific CD8^+^ T cells secreting INF-γ or CD69 expression between mild/moderate patients and severe/critical patients at acute stage of the disease (Mann-Whitney *U* test). *P* values of ≤0.05 were considered statistically significant. IFN, interferon; SEB, Staphylococcal enterotoxin B.

**Figure 6:**
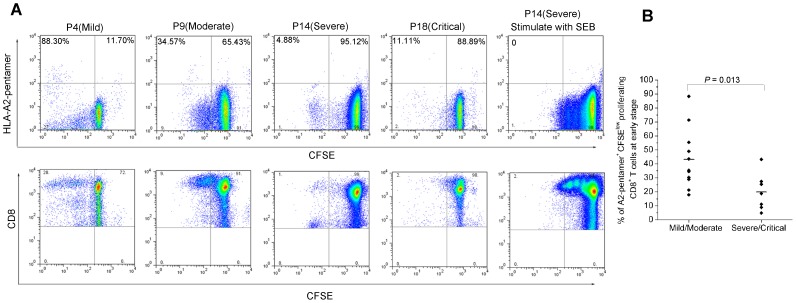
The proliferation capacity of HTNV nucleoprotein epitope-specific CD8^+^ T cells at acute stage of HFRS. (A) The PBMCs of four HLA-A2^+^ patients with different severities stimulated with the epitope aa129–aa137 were detected. FACS contour plots were gated on CD3^+^ CD8^+^ cells. The proliferative potential of the epitope-specific CD8^+^ pentamer^+^ T cells was shown in the upper left quadrants in the *upper lane* of the figure suggesting the loss of CFSE in the dividing CD8^+^ T cells. The *lower lane* showed the proliferative capacity of the two CD8^+^ T-cell subsets. (B) The comparison of the frequency of HLA-A2-pentamer^+^ CFSE^low^ proliferating CD8^+^ T cells between mild/moderate patients and severe/critical patients at acute stage of the disease (Mann-Whitney *U* test). *P* values of ≤0.05 were considered statistically significant. CFSE, 5, 6-carboxyf luorescein succinimidyl ester. SEB, Staphylococcal enterotoxin B.

Interestingly, it seemed that there were two subsets of CD8^+^ T cells in PBMCs with high or low mean fluorescence intensity, both of which could respond to the specific epitopes presented by the pentamers (Figure 3). The further staining showed that both CD8^hi^ and CD8^lo^ T cells were CD8αβ heterodimer expression (Figure S1). The much higher ratio of CD8^lo^/CD8^hi^ T cell-numbers in the acute stage would decline at late stage of HFRS. Both the CD8^lo^ and CD8^hi^ T cell subsets could produce IFN-γ when stimulated with specific epitopes (Figure 5A). However, a stronger proliferative capacity of epitope-specific CD8^hi^ T cell subset than that in the CD8^lo^ T cell subset at the acute stage was observed, as shown by the pentamer^+^ cells in the upper left quadrants of the dot plots (Figure 6A *lower panel*).

The quantity of epitope-specific CD8^+^ CD69^+^ T cells producing IFN-γ at acute and late time points were performed in Figure 5A, where the examples of four HLA-A2^+^ patients with indicated severity showed that a decline tendency could also be found in the kinetics of IFN-γ quantity (*upper panel*), and the peptide-induced up-regulation of CD69 was detectable at both time points in the PBMCs of the patients (*lower panel*). The frequency of IFN-γ produced by the CD8^+^ CD69^+^ T cells was much higher in patients with mild/moderate severity than in severe/critical ones at the acute stage of HFRS (Mann–Whitney *U* test, for aa129–aa137: *P* = 0.037; for aa131–aa139: *P* = 0.043) (Figure 5B–5C). Despite the low number of total activated epitope-specific CD8^+^ T cells, a high proliferative potential of the pentamer^+^ CD8^+^ T cells was observed (Figure 6A). For epitope aa129–aa137, the proliferative capacity of the epitope-specific CD8^+^ T cells was stronger in mild/moderate patients than that in severe/critical patients (Mann–Whitney *U* test, *P* = 0.013) (Figure 6B).

## Discussion

In this study, we defined five novel HTNV-NP CTL epitopes restricted by the major HLA class I molecules in Chinese Han population; we provided, for the first time, a quantification of the HTNV epitope-specific IFN-γ–secreting CD8^+^ T cells in HFRS patients; we analyzed the kinetics changing, the activation, and proliferation capacity of the epitope-specific CD8^+^ T cells and evaluated the associations between the epitope-specific CD8^+^ T-cell frequencies and the different outcomes of the HFRS severity.

Generally, the residues on these novel epitopes we defined are well conserved, especially among the Old World Hantaviruses that cause HFRS. Specifically, the HTNV and Dobrava virus share the identical sequences of three epitopes we defined, although the endemic areas of these two viruses are far from each other. We speculate that the sequences of well-conserved epitopes may locus mainly at the conserved region among the genus Hantaviruses. Particularly, a well-conserved epitope aa131–aa139 with little variation in the HLA anchor residues restricted by HLA-B35 was defined, in accordance with both the sequence and HLA restriction of the epitope identified on the NP of the SNV and Andes virus previously [31], [32], which make our results more credible.

The quantitation of peptide-specific CD8^+^ T cells demonstrated that the epitopes we defined could bind to the corresponding HLA class I molecules to form the HLA-peptide complex, which could stimulate epitope-specific CD8^+^ T-lymphocyte responses. Compared with our previous general study [25], we focused on the HLA restricted single epitope-specific CD8^+^ T-cell responses and found that there was an obvious inverse association between the magnitude of the epitope-specific CD8^+^ T-cell responses in the acute stage and the severity degree of the HFRS, which was in accordance with our recently findings that at the acute stage of disease, patients in severe/critical group were found to have higher viral loads than those in mild/moderate group [33], suggesting that functional HTNV epitope-specific CD8^+^ T cells at the acute stage of HFRS would be important for the clearance of the virus. Furthermore, we found there was a tendency of decline of the epitope-specific CD8^+^ T-cell frequencies from acute stage to convalescence, which was inconsistent with our previous results that the gradually increased frequencies of HTNV NP-specific T-cell responses during the course of the disease was found with IFN-γ ELISPOT assay [25]. There are several critical methodological differences between these two studies. However, the Finnish group found when studying HFRS caused by PUUV that IFN-γ ELISPOT assays using PBMCs obtained in the acute stage of the disease hugely underestimated the frequency of peptide-specific T cells compared to HLA/peptide tetramer staining probably due to activation induced apoptosis of these peptide-specific T cells during incubation with the peptides for the assay [20]. Thus the conclusions would be taken by caution in our previous study [34]. The decreased frequencies of HTNV-NP epitope-specific CD8^+^ T cells were consistent with the findings that HTNV RNA load could only be detected in plasma of HFRS patients in febrile/hypotensive and oliguric stage and gradually declined to an undetectable level with the progress of the disease [33].

Additional analysis found that the levels of single epitope-specific CD8^+^ T-cell response at the early stage of the disease associated with clinical parameters. The peak level of serum creatinine and the nadir of platelet counts during the course of HFRS usually could provide a better prediction of disease severity. The higher frequency of the epitope-specific CD8^+^ T cells at the acute stage of the patients, the lower peak level of serum creatinine and the higher nadir of platelet counts during hospitalization, which indicated that the level of the HTNV-NP epitope-specific CD8^+^ T-cell responses at the acute stage could predict the different outcomes of the disease. However, the association between the NP-specific CD8^+^ T-cell response and the disease severity still needs to be investigated, since the major immunodominant structure proteins of HTNV that could induce the CD8^+^ T-cell response is uncertain.

Furthermore, the studies conducted on the function of the virus-specific CD8^+^ T-cell responses in different viruses, usually with specific HLA restrictions, are currently controversial [22], [35], [36]. Similar with our finding, a study conducted on the Andes virus-infected patients found that among the HLA-B35-positive patients, mild disease outcome seemed to be associated with stronger responses toward the Gn-carboxyterminus than that in patients with severe HPS [32]. Whereas the study on SNV showed that the intense responses of the epitope-specific CD8^+^ T cells restricted by HLA-B3501 contribute to the severe outcome of HPS [23]. Therefore, the different HLA allele restrictions and different viruses would be important factors influencing the T cell responses and the outcome of the diseases that should be taken into account in such anti-virus cellular immune response studies.

Another finding from the current data is that the frequency of the HTNV epitope-specific pentamer^+^ CD8^+^ T cells was low, regardless of the severity of the HFRS patients. It is not surprising for the result because only the NP epitope-specific CTL response was detected here. The low frequencies may be due to the relatively low contribution of NP-directed T-cell responses, in comparison to the surface glycoprotein, which is the other important structure antigen of HTNV and can induce much stronger CTL responses than NP, as indicated in the studies of other Hantaviruses [23], [32], especially the report on SNV infections, in which the frequency of epitope-specific CD8^+^ T cells has been reported to be as high as 44.2% [23]. In fact, during the millennia of severe Hantavirus infections, persons with susceptible HLA alleles may have been eliminated from certain human populations, thus skewing the distribution of alleles in the population. Since the HLA-A2 and -B35 in our present study are the major alleles in Chinese Han population, but not the alleles with susceptibility in HFRS patients [37], [38], whereas the HLA-B3501 allele is a risk factor for severe HPS and relatively common allele among HPS patients induced by the New World Hantaviruses [23], [39], we speculate that the different genetic susceptibility should also be taken into account to explain the differences of the frequency of T-cell response among Hantaviruses.

Although our data demonstrated that HTNV-specific CD8^+^ T-cell responses directed against the individual epitope could be detected with the pentamer staining, a high proportion of subpopulation CD8^lo^ T cells presented in the early stage of HTNV infection. It is interesting to evaluate whether both these subsets of CD8^+^ T cells have similar behaviors that contribute to the anti-viral effects. According to the studies on other virus infectious diseases, polyfunctional CD8^+^ T cells may play different roles in the pathogenesis of the disease [40], [41]. Compared with epitope-specific CD8^hi^ T cell subset, the CD8^lo^ T cells with the similar ability to secrete IFN-γ and the milder proliferation capability at the acute stage of the disease might be represented with the same peptide specificity, but with a poly-cytokines secretion profile or a variety of phenotypes, which might contribute to the clearance of the virus and the milder severity of the disease. Due to limitations in the PBMC samples, we were unable to further test for differential cytokine secretion profiles for both the subsets and the cause of this CD8^lo^ T cell subset, but we propose that both of them would have a similar effect function with more or less distinction that need to be confirmed next. An overall analysis revealed that the epitope-specific CD8^+^ T cells up-regulated CD69 after exposure to peptides, representing an activated phenotype and showed obvious capacity to secret IFN-γ and proliferation in acute infection. Similar with many other virus infectious diseases [42], [43], epitope-specific CD8^+^ T-cell proliferation generally correlated with effective immunity, as the capacity of proliferation was slight in severe/critical patients, which represents a further step in the understanding of the impairment of CD8^+^ T cell functions in severe/critical HFRS patients.

In summary, such a thorough study on the identification of HLA Class I-T cell epitopes of HTNV and evaluation of the function of single epitope-specific CD8^+^ T-cell response on the clinical outcomes have not been reported before in the field of HTNV investigation. Our results might provide new insights into understanding the relationship between single epitope-specific CD8^+^ T-cell response, CD8^+^ T-cell functional characters, and disease control in acute zoonotic HTNV infections in humans, which could be a rationale to explore immunotherapy as an adjunctive therapy in people with HFRS and will help to speed up the novel vaccine design process against the HTNV infection.

Since the first step toward a peptide vaccine is epitope mapping of the HTNV structure proteins according to the most frequent HLA alleles in Chinese Han population, the only research on the T-cell epitopes and their responses to NP is just a fraction of the research work. The epitopes and the immunogenicity of the HTNV glycoproteins, which could elicit even higher detectable T-cell responses in HFRS patients, are still needed investigation to make a comprehensive explanation of the cellular immunity functions after HTNV infection. The combination of the studies on both HTNV-NP and glycoprotein will be more valuable and helpful to understand the immune interaction between the HTNV and the host. Besides, whether our results could be generalized to T-cell responses to peptides presented by other HLA alleles and whether broadening or magnifying the CD8^+^ T-cell response in patients with severe infection is possible still need to be investigated.

## Supporting Information

Figure S1 The PBMCs of the four HLA-A2^+^ patients, including mild patient P4, moderate patient P9, severe patient P14, and critical patient P18, were gated on CD3^+^ T cells and then stained with CD8α and CD8β to determine the CD8αβ T cell subset.
(TIF)

Table S1 The characteristics of the hemorrhagic fever with renal syndrome patients and the frequencies of the phenotypes of T cells at the acute stage of the disease.
(DOC)

Text S1 Procedures and investigations undertaken in Methods.
(DOC)
