# Supplementary figures and images for: HLA-A2 and B35 Restricted Hantaan Virus Nucleoprotein CD8+ T-Cell Epitope-Specific Immune Response Correlates with Milder Disease in Hemorrhagic Fever with Renal Syndrome
Source: PLoS Negl Trop Dis. 2013 Feb 28;7(2):e2076. doi: 10.1371/journal.pntd.0002076 (PMC3585118; doi:10.1371/journal.pntd.0002076)

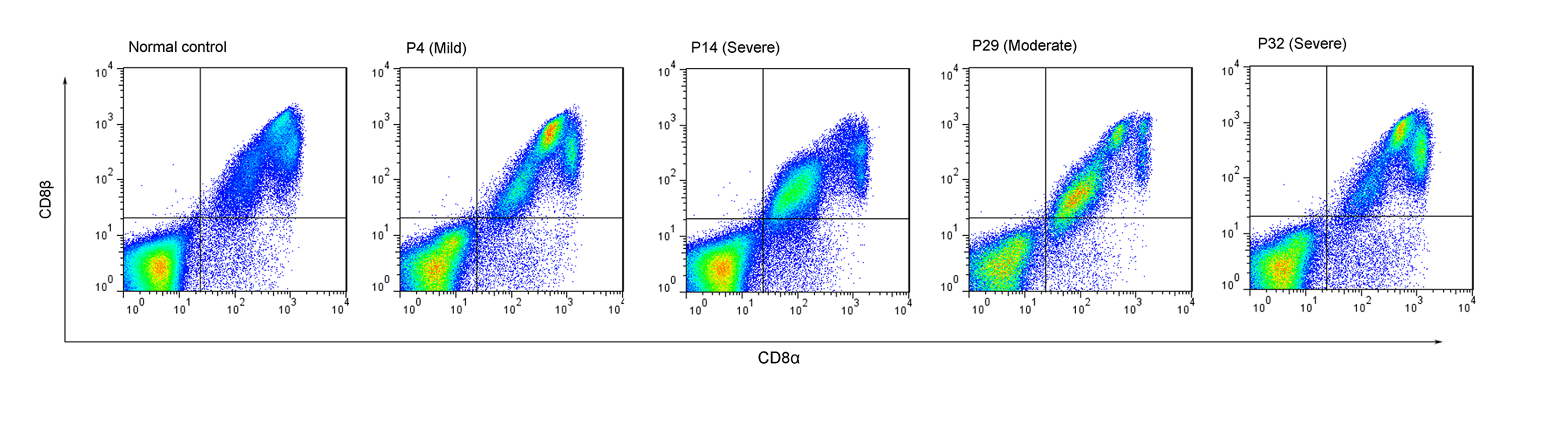

Supplement: Figure S1 — The PBMCs of the four HLA-A2+ patients, including mild patient P4, moderate patient P9, severe patient P14, and critical patient P18, were gated on CD3+ T cells and then stained with CD8α and CD8β to determine the CD8αβ T cell subset. (TIF) [file pntd.0002076.s001.tif]
