# Supplementary material for: HLA-A2 and B35 Restricted Hantaan Virus Nucleoprotein CD8+ T-Cell Epitope-Specific Immune Response Correlates with Milder Disease in Hemorrhagic Fever with Renal Syndrome
Source: PLoS Negl Trop Dis. 2013 Feb 28;7(2):e2076. doi: 10.1371/journal.pntd.0002076 (PMC3585118; doi:10.1371/journal.pntd.0002076)
Supplement: Table S1 — The characteristics of the hemorrhagic fever with renal syndrome patients and the frequencies of the phenotypes of T cells at the acute stage of the disease. (DOC) [file pntd.0002076.s002.doc]

**Table S1**. The characteristics of the hemorrhagic fever with renal syndrome patients and the frequencies of the phenotypes of T cells at the acute stage of the disease.

| **Peptide tested** | **Patient ID** | **Severity** | **Sex** | **Age(y)** | **The days of illness onset when detection at acute stage** | **Maximun serum creatinine (μmol/l)** | **Nadir platelet counts (*1000cells/μl)** | **Effector phenotype of T cell at acute stage (%)^b^** | | | |
| --- | --- | --- | --- | --- | --- | --- | --- | --- | --- | --- | --- |
|  |  |  |  |  |  |  |  | **Pentamer^+^ CD8^+^** | **CD8^+^ IFN-γ^+^** | **CD69^+^ IFN-γ^+^** | **Pentamer^+^ CFSE^-^** |
|  |  |  |  |  |  |  |  |  |  |  |  |
| A*02 restricted aa129-aa137 | P1 | Moderate | M | 56 | 6 | 439.3 | 12 | 0.070 | 0.066 | 0.072 | 30.30 |
|  | P2 | Moderate | M | 34 | 8 | 88.6 | 49 | no sample^a^ |  |  |  |
|  | P3 | Moderate | F | 44 | 5 | 123.6 | 67 | 0.010 | 0.010 | 0.015 | 21.30 |
|  | P4 | Mild | M | 51 | 8 | 102 | 167 | 0.700 | 0.078 | 0.065 | 88.30 |
|  | P5 | Moderate | F | 34 | 4 | 408 | 89 | 0.210 | 0.065 | 0.039 | 43.51 |
|  | P6 | Moderate | M | 29 | 7 | 606 | 69 | 0.168 | 0.042 | 0.045 | 49.00 |
|  | P7 | Moderate | M | 57 | 7 | 301.1 | 80 | 0.430 | 0.078 | 0.032 | 28.90 |
|  | P8 | Moderate | M | 30 | 8 | 69.7 | 105 | 0.370 | 0.100 | 0.055 | 55.38 |
|  | P9 | Moderate | M | 25 | 8 | 407 | 56 | 0.360 | 0.094 | 0.157 | 34.57 |
|  | P10 | Mild | M | 38 | 4 | 602 | 63 | 0.250 | 0.078 | 0.076 | 35.42 |
|  | P11 | Mild | M | 46 | 6 | 205.6 | 13 | 0.220 | 0.062 | 0.068 | 71.35 |
|  | P12 | Moderate | M | 45 | 4 | 304.8 | 15 | 0.125 | 0.040 | 0.051 | 17.86 |
|  | P13 | Severe | M | 57 | 8 | 748 | 9 | 0.051 | 0.030 | 0.022 | 25.40 |
|  | P14 | Severe | M | 60 | 5 | 369 | 50 | 0.051 | 0.049 | 0.024 | 4.88 |
|  | P15 | Critical | M | 31 | 5 | 358.7 | 42 | no sample^a^ |  |  |  |
|  | P16 | Severe | M | 23 | 6 | 688 | 12 | 0.066 | 0.032 | 0.050 | 43.20 |
|  | P17 | Severe | M | 66 | 6 | 650 | 15 | 0.084 | 0.045 | 0.038 | 18.75 |
|  | P18 | Critical | F | 70 | 6 | 593.8 | 128 | 0.066 | 0.035 | 0.045 | 11.11 |
|  | P19 | Severe | M | 56 | 4 | 429.5 | 56 | 0.100 | 0.062 | 0.048 | 27.40 |
|  | P20 | Severe | M | 50 | 7 | 798.35 | 11 | 0.029 | 0.010 | 0.012 | 8.88 |
|  | P21 | Critical | F | 19 | 6 | 889.2 | 21 | N.D. |  |  |  |
|  | P22 | Critical | M | 32 | 3 | 629.81 | 13 | N.D. |  |  |  |
|  | P23 | Critical | F | 49 | 6 | 712.9 | 9 | N.D. |  |  |  |
|  | P24 | Critical | F | 64 | 8 | 1098 | 14 | N.D. |  |  |  |
|  | P25 | Critical | F | 36 | 8 | 938.7 | 21 | N.D. |  |  |  |
| B*35 restricted aa131-aa139 | P26 | Moderate | M | 55 | 8 | 593.89 | 56 | 0.097 | 0.056 | 0.056 | no sample^a^ |
|  | P27 | Mild | M | 41 | 6 | 530 | 24 | 0.185 | 0.088 | 0.075 | 34.78 |
|  | P28 | Moderate | M | 39 | 4 | 674 | 21 | 0.133 | 0.04 | 0.066 | 78.90 |
|  | P29 | Moderate | F | 40 | 3 | 480 | 28 | 0.166 | 0.042 | 0.04 | no sample^a^ |
|  | P30 | Critical | F | 61 | 7 | 668.3 | 16 | 0.054 | 0.036 | 0.042 | no sample^a^ |
|  | P31 | Severe | M | 33 | 7 | 660.4 | 12 | 0.042 | 0.027 | 0.033 | no sample^a^ |
|  | P32 | Severe | M | 34 | 6 | 879.9 | 19 | 0.033 | 0.02 | 0.010 | 16.75 |
|  | P33 | Critical | F | 51 | 8 | 846.5 | 10 | 0.018 | 0.01 | 0.016 | no sample^a^ |

IFN, interferon; CFSE, 5,6-carboxyf luorescein succinimidyl ester; M, male; F, female; N.D., not detected.

^a^ No sample, there was not enough peripheral blood mononuclear cells for detection.

^b^ The percentages given were indicated as the percentage of the live CD3^+^ lymphocytes.
