# Supplementary material for: HLA-A2 and B35 Restricted Hantaan Virus Nucleoprotein CD8+ T-Cell Epitope-Specific Immune Response Correlates with Milder Disease in Hemorrhagic Fever with Renal Syndrome
Source: PLoS Negl Trop Dis. 2013 Feb 28;7(2):e2076. doi: 10.1371/journal.pntd.0002076 (PMC3585118; doi:10.1371/journal.pntd.0002076)
Supplement: Text S1 — Procedures and investigations undertaken in Methods. (DOC) [file pntd.0002076.s003.doc]

**Procedures and Investigations undertaken as follow:**

Sample Collection

The blood samples were collected during hospitalization from the onset of fever symptom. Peripheral blood mononuclear cells (PBMCs) were isolated from EDTA-treated blood samples by gradient centrifugation on Ficoll–Hypaque (Sigma-Aldrich, MO) and cryopreserved until use.

Synthetic Peptides

Seventy 15-mer peptides, overlapping by 9 residues, spanning the nucleoprotein (NP) sequence of the Hantaan virus (HTNV) strain 76-118 (GenBank accession no. M14626) were synthesized and pooled as previously described [27]. To define the optimal peptide sequence for CD8^+^ T-cell epitopes, N- and C-terminal truncated peptides were also synthesized for each CD8^+^ T-cell responsive 15-mer peptide. All the peptides were purified (90% purity) and stored at a concentration of 1mM at –70℃ and avoided [repeated freeze-thaw](http://dict.cnki.net/dict_result.aspx?searchword=反复冻融&tjType=sentence&style=&t=repeated+freeze-thaw).

HLA Typing

The PBMCs of the hemorrhagic fever with renal syndrome (HFRS) patients were transformed by the Epstein-Barr virus (EBV) (using a supernatant of the cultured B958 cell line) into EBV-B cells [27]. The DNA was extracted from EBV-B cells using DAN^zol^ (Invitrogen, California). HLA typing was performed using standard molecular techniques [43].

In vitro peptide-specific expansion of CD8^+^ T cells

The CD8^+^ T cells were positively isolated from the PBMCs of HFRS patients using the CD8^+^ T-cell Positive Isolation Kit (Invitrogen Dynal AS, Norway) according to the manufacturer’s instructions. More than 90% of the CD8^+^ T-cell isolation was confirmed by a flow cytometric analysis. Then, 5 × 10^5^ isolated CD8^+^ T cells were stimulated with 1 × 10^6^ irradiated autologous CD8^+^ T-cell-depleted PBMCs, serving as antigen-presenting cells, and were pre-pulsed with 10 μM of the single 9-mer peptide for 2 h, in 200 μl complete medium (RPMI 1640 containing 10% fetal bovine serum and 1% penicillin/streptomycin) in a 96-well plate. Overall, 20 U/ml IL-2 (Roche Molecular Biochemicals, Germany) and 20 ng/ml IL-7 (R&D Systems, MN) were added for eight hours, and, subsequently, cultures were fed every three days with a medium containing IL-2 (10 U/ml) for 10 to 14 days before the cells were harvested.

Ex vivo Interferon-γ (IFN-γ) enzyme-linked immunospot (ELISPOT) Assays

The cryopreserved PBMCs of HFRS patients were thawed and rested overnight before being placed in the ELISPOT plates at 2 × 10^5^ cells/well. The PBMCs were screened for 24 hours with ten pooled overlapping 15-mer peptides, in which each peptide had a final concentration of 10 μM. Each peptide pool comprised of seven individual peptides. The single-positive 15-mer peptide was then identified from the positive-response peptide pools. The recognition of CD4^+^ T-cell responsive 15-mer epitopes or CD8^+^ T-cell responsive 9-mer epitopes was evaluated by the ELISPOT assay (Mabtech, Germany) using CD8^+^ T-cell-depleted or CD4^+^ T-cell-depleted PBMCs. HLA restriction of the CD8^+^ T-cell response to the single 9-mer epitope was defined using an *in vitro* epitope-specific expansion of CD8^+^ T cells as effector cells (5×10^4^ cells/well) and using EBV-B cell lines sharing partly HLA class I molecules pulsed with a particular peptide as antigen presenting cells (5×10^4^ cells/well) followed by the standard ELISPOT assay. Phytohemagglutinin (10 μg/ml, Sigma-Aldrich, MO) was always included as a positive control, and no peptide or EBV-B cells without peptide was employed as a background control for each individual test. The automated ELISPOT reader (Cellular Technology Limited, USA) was used to count the spots. The positive response was defined as at least 50 spot forming cells (SFCs)/10^6^ input cells and exceeding thrice above the background response after subtracting the number of spots in the background controls from that in the stimulated samples.

HLA class I pentamer Staining

Two PE-labeled HLA-A2 and -B35 pentamers refolded with the CD8^+^ T-cell 9-mer epitopes FVVPILLKA and VPILLKALY, respectively were customized from Proimmune (Oxford, UK). The PBMCs of the patients or the healthy controls were stained with PE-labeled HLA class I pentamer for 10 min incubation at room temperature, and subsequently stained with the mouse monoclonal antibodies to CD3 APC (clone UCHT1) and CD8 FITC (RPA-T8) (BD Pharmingen) for 20 min on ice. 7-AAD (eBioscience) was added to each tube in order to discriminate between live and dead cells before acquisition of the cells. A minimum of 500,000 total cells were acquired and gated on the live CD3^+^ cells. The CD8^+^ pentamer^+^ T-cell gate was set by the isotype control (BD Pharmingen) staining, and the cutoffs for positive staining were set by staining with an irrelevant pentamer. To determine the CD8^+^ T cell subsets, the PBMCs of the patients were gated on CD3^+^ T cells and then stained with anti-CD8α FITC (RPA-T8) and anti-CD8β PE (2STB.5H7) (BD Pharmingen). Compensation controls were checked regularly and individually determined for each experimental setup.

Intracellular Cytokine Staining

The 2 × 10^6^ PBMCs were stimulated with 5 × 10^5^ peptide-pulsed autologous EBV-B cells (10 μM for 1 h) in the presence of co-stimulatory antibodies anti-CD28 and anti-CD49d (1 μg/ml; BD Biosciences) for 1 h. Brefeldin A (10 μg/ml; Sigma-Aldrich) and Monensin (0.7 μl/ml; BD Biosciences) were added for further 5 h incubation. The staphylococcal enterotoxin B (SEB, 200 ng/ml, Sigma-Aldrich, MO) was added as a positive control, and an unstimulated (complete medium only) was used as a negative control. Then the anti-CD3 FITC (UCHT1) and anti-CD8 APC (RPA-T8) (BD Pharmingen) were added for 30 minutes at 4℃ in the dark, followed by fixing and permeabilizing for 20 minutes in the dark at 4℃ with the Cytofix/Cytoperm kit (BD Biosciences) and the anti-IFN-γ PE (4S.B3) and anti-CD69 PE-Cy5 (FN50) (BD Pharmingen) staining for 45 min at 4℃ in the dark. Between 150,000 and 500,000 cells were collected for each tube. The cytokine response was considered positive when the percentage of the responding CD8^+^ subset was greater than 0.01% after background subtraction.

Ex vivo Proliferation Assay

The 2 × 10^7^/ml PBMCs were incubated for 15 min with 10 μM 5, 6-carboxyf luorescein succinimidyl ester (CFSE, Molecular Probes, OR) in PBS at 37℃. CFSE was then neutralized by fetal bovine serum, and PBMCs were cultured with particular HTNV-NP 9-mer peptide (10 μM). The SEB (200 ng/ml) stimulation served as a positive control. After 2 days, 10% exogenous IL-2 was added. At day 7, the cells were harvested and stained with the PE-labeled pentamer, CD8 APC (RPA-T8) and 7-AAD [42]. About 300,000 to 800,000 cells were acquired on a FACScalibur (BD Immunocytometry Systems, California). Data analysis was performed using FlowJo 8.8.2 software (TreeStar, CA).
